# Small-cell carcinoma of the cervix with acute-onset psychotic symptoms associated with clinically diagnosed ectopic ACTH production: a case report

**DOI:** 10.3389/fonc.2026.1684861

**Published:** 2026-03-18

**Authors:** Kanako Ozaki, Junya Fujino, Kaoru Niimi, Kuniyuki Iwata-Endo, Naoki Otsuka, Tomoko Kobayashi, Kosuke Yoshida, Kazumasa Mogi, Masato Yoshihara, Yukari Nagao, Satoshi Tamauchi, Akira Yokoi, Nobuhisa Yoshikawa, Hiroaki Kajiyama

**Affiliations:** 1 Department of Obstetrics and Gynecology, Nagoya University Graduate School of Medicine, Nagoya, Japan; 2 Department of Psychiatry, Nagoya University Graduate School of Medicine, Nagoya, Japan; 3 Department of Endocrinology and Diabetes, Nagoya University Graduate School of Medicine, Nagoya, Japan

**Keywords:** case report, Cushing’s syndrome, ectopic ACTH producing tumor, psychiatric symptoms, small-cell carcinoma of the cervix

## Abstract

Small-cell carcinoma of the cervix (SCCC) is a rare and highly aggressive histological subtype of cervical cancer, associated with poor prognosis. SCCC is histologically classified as a neuroendocrine tumor and has the potential to produce ectopic hormones, leading to various paraneoplastic syndromes. This report is a rare case of recurrent SCCC presenting with psychiatric symptoms due to endogenous Cushing’s syndrome caused by ectopic adrenocorticotropic hormone (ACTH) production. The patient initially developed mood and behavioral disturbances as the disease progressed, leading to hospitalization under the suspicion of a primary psychiatric disorder. However, further evaluation, prompted by the discovery of severe hypokalemia, revealed Cushing’s syndrome associated with clinically diagnosed ectopic ACTH production in the setting of recurrent disease. Her psychiatric symptoms rapidly remitted following the administration of a cortisol synthesis inhibitor. This case highlights the importance of considering endocrine disorders as potential causes of psychiatric manifestations in patients with cancer, particularly those with neuroendocrine tumors such as SCCC. Acute and marked elevation of endogenous cortisol can induce distinct psychiatric symptoms, such as manic features and grandiose delusions, that often respond better to endocrine treatment aimed at normalizing cortisol levels rather than to antipsychotic therapy alone. Clinicians should be aware of this rare but important clinical presentation as timely diagnosis and management can improve patient outcomes.

## 1 Introduction

Small-cell carcinoma of the cervix (SCCC) is a rare histological subtype, accounting for less than 5% of cervical cancers, and is associated with treatment resistance and poor prognosis. It is classified as a neuroendocrine tumor and presents various symptoms owing to the production of ectopic hormones. For example, it can cause syndrome of inappropriate secretion of antidiuretic hormone (SIADH) and Cushing’s syndrome. Endogenous Cushing’s syndrome is a rare endocrine disorder characterized by excessive cortisol secretion. It is associated with psychiatric illness and neurocognitive impairment. Specifically, an acute increase in cortisol levels can precipitate a manic or hallucinatory–delusional state (1). Here, we present a case of recurrent SCCC with liver metastases 1 year after the original diagnosis, with psychiatric symptoms of an ectopic adrenocorticotropic hormone (ACTH)-producing tumor.

## 2 Case description

**Figure 1:**
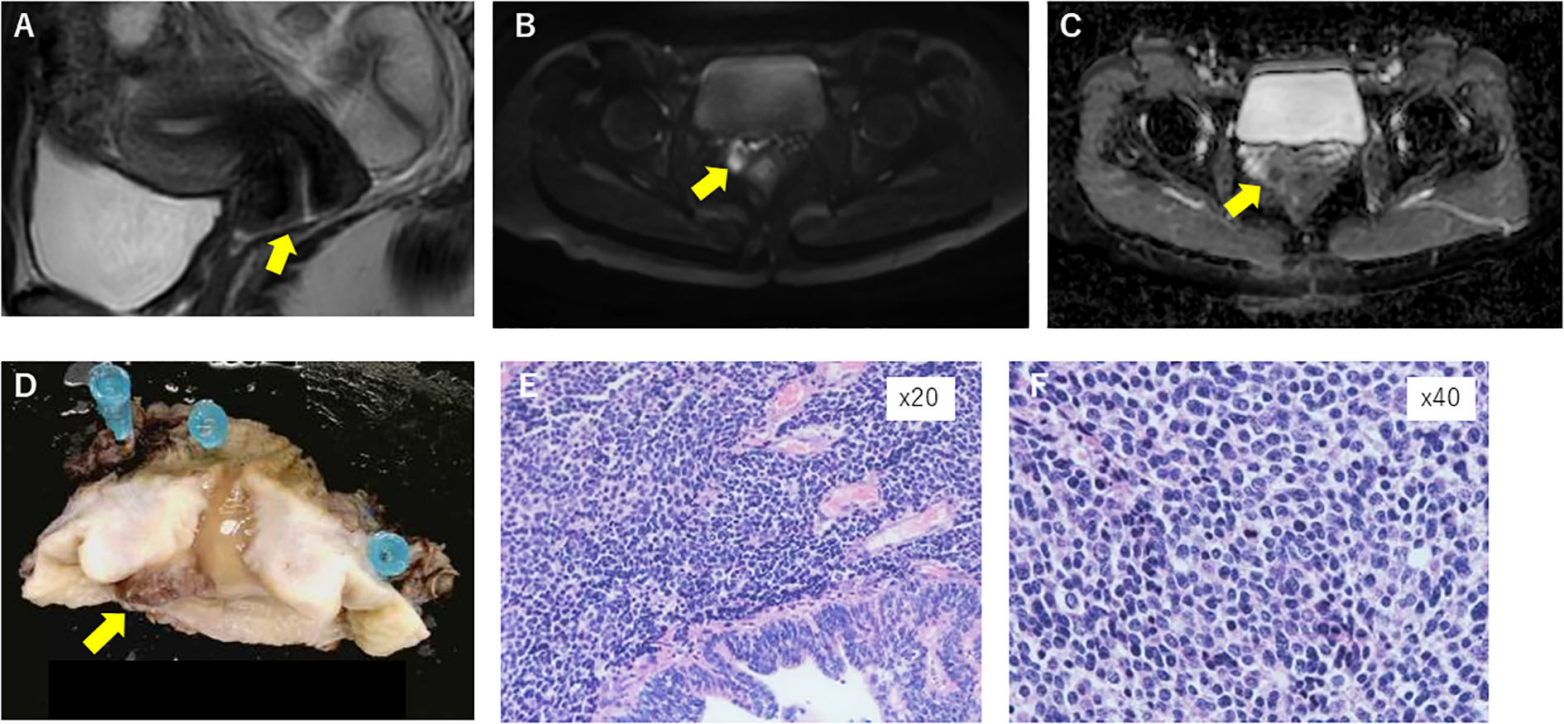
Contrast MRI before operation revealed a 10-mm mass in front of the cervix **(A)**, presenting as a high diffusion-weighted image (DWI) **(B)** with a low apparent diffusion coefficient (ADC) **(C)**. Macroscopic and microscopic appearance of the surgical specimen **(D)**, which presents combined small-cell neuroendocrine and adenocarcinoma in hematoxylin–eosin staining (**E**; ×20); pT1b1, pN0, and vascular invasion was positive (**F**; ×40).

A 39-year-old Japanese woman, who was married and not gravida, received infertility treatment at a local clinic. A cervical mass was found, and histopathological examination revealed combined adenocarcinoma and small-cell neuroendocrine carcinoma. Pelvic examination at our hospital revealed a well-defined pinky finger mass. Contrast-enhanced magnetic resonance imaging (MRI) revealed a 10-mm mass located in front of the cervix, which presented as a high diffusion-weighted image (DWI) with a low apparent diffusion coefficient (ADC) (**Figures 1A–C**). Based on these results, the patient was diagnosed with cervical cancer.

We recommended that the patient receive a radical hysterectomy because small-cell carcinoma has a high malignancy rate and poor prognosis; however, the patient had a strong desire to preserve fertility. After thorough counseling and informed consent, the patient underwent radical trachelectomy. Histopathological examination revealed a combination of small-cell neuroendocrine tumors and adenocarcinoma, pT1b1 and pN0 (**Figures 1D–F**). Lymphovascular space invasion was identified. Combined chemoradiation therapy (CCRT) was recommended because of the presence of lymphovascular space invasion and the aggressive nature of small-cell neuroendocrine carcinoma. Despite the diagnosis of small-cell neuroendocrine carcinoma and lymphovascular space invasion, the patient strongly desired fertility preservation. The aggressive biological behavior of SCCC, its poor prognosis, and the lack of established evidence supporting fertility-sparing surgery were thoroughly discussed with the patient. After multidisciplinary consultation and obtaining informed consent acknowledging the potential oncologic risks, radical trachelectomy followed by chemotherapy was selected under a policy of strict postoperative surveillance.

**Figure 2:**
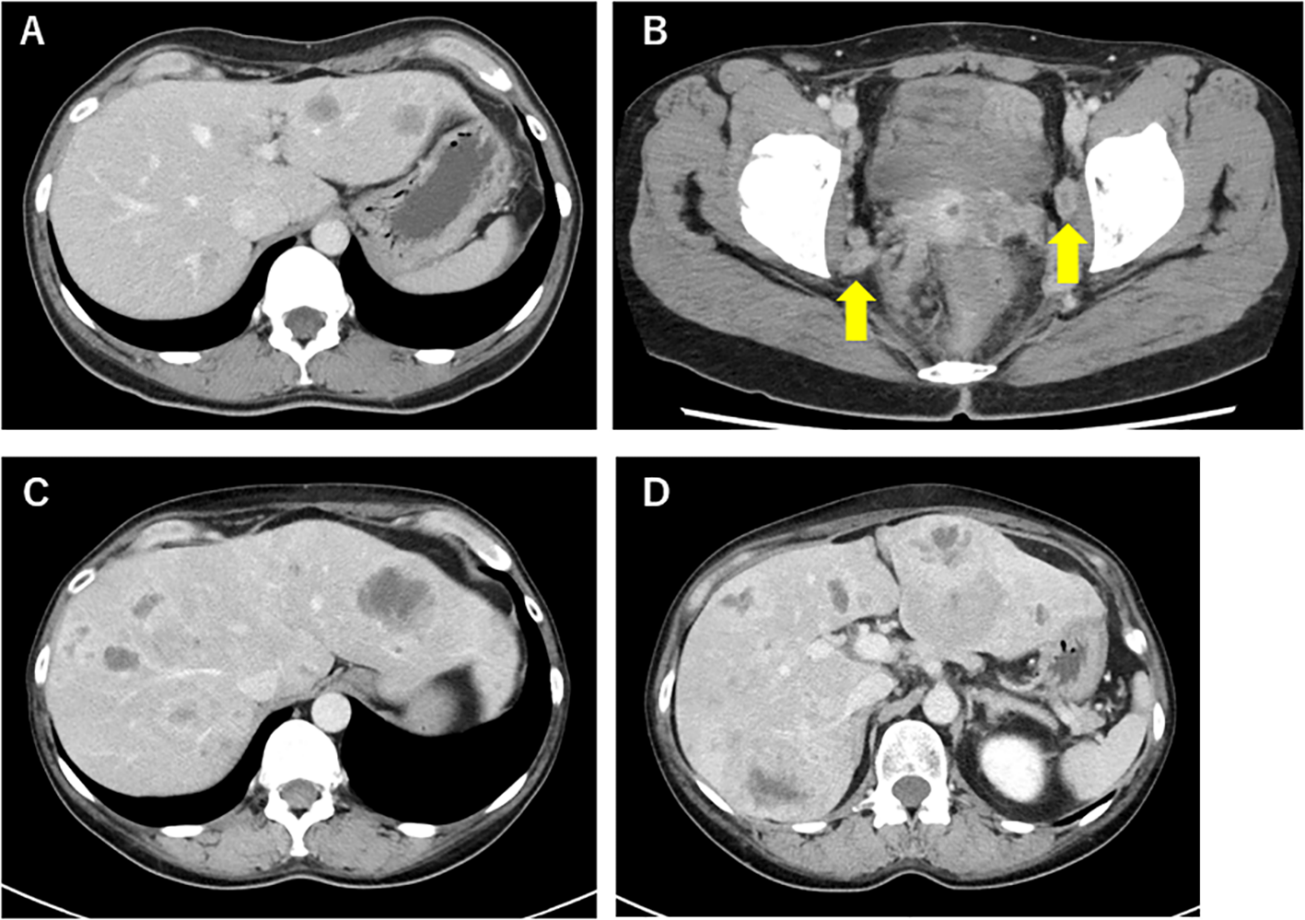
Abdomen CT scan 5 months after her first chemotherapy, which revealed multiple metastases of the liver **(A)** and lymph nodes **(B)**. Abdomen CT scan for psychiatric symptoms (Day 1). This revealed an increase in the size of liver metastases **(C, D)**.

After three courses of etoposide and cisplatin therapy, no residual lesions were observed on computed tomography (CT) scans. However, CT 5 months later revealed multiple metastases to the lymph nodes and liver (**Figure 2A, B**), and the tumor marker neural-specific enolase (NSE) rose to 38.8 ng/mL.

**Figure 3:**
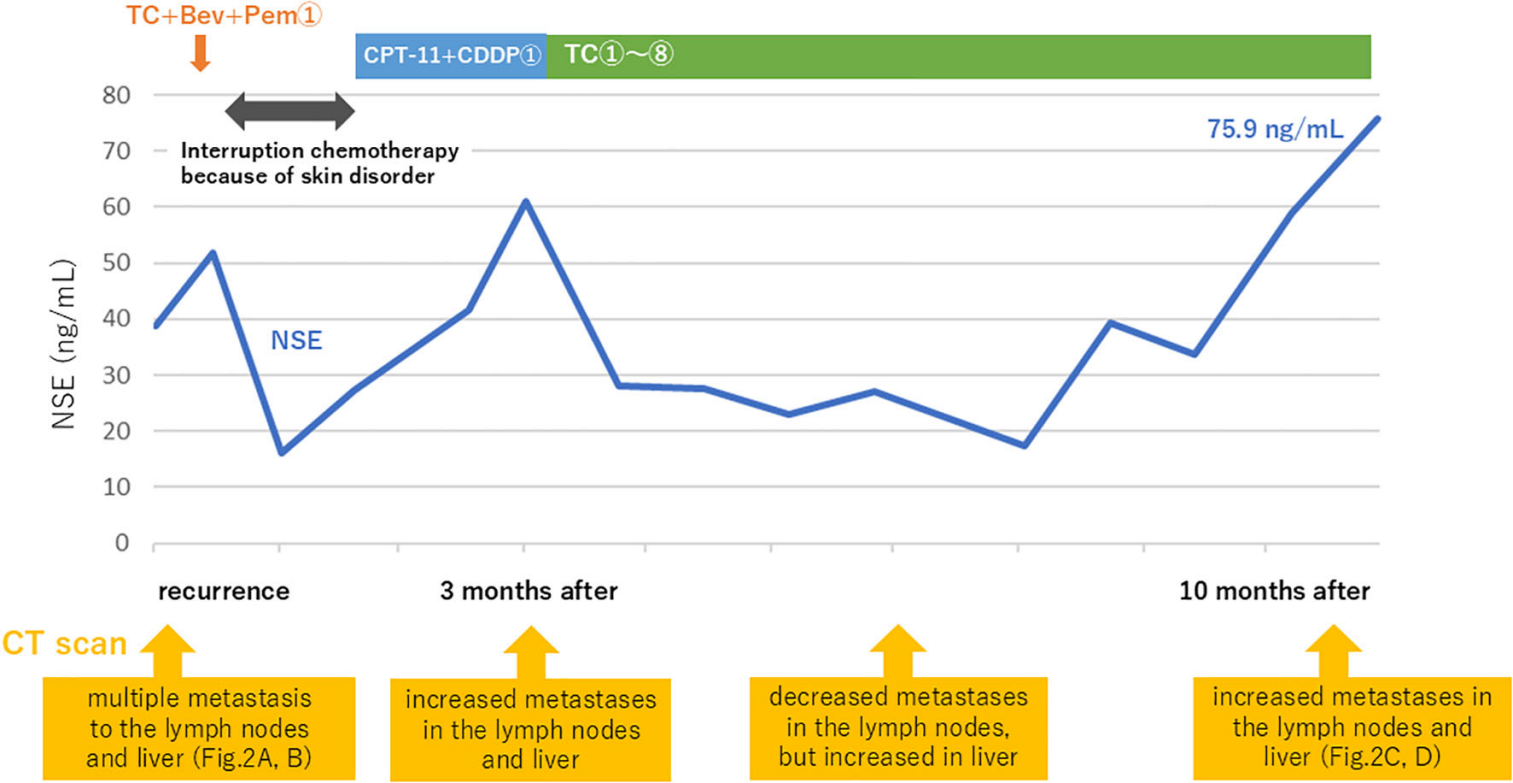
Treatment course after recurrence, including changes in serum NSE levels and CT findings.

As treatment for recurrence, paclitaxel and carboplatin in combination with bevacizumab and pembrolizumab therapy was administered, which was discontinued after a single course because of a skin disorder suspected to be an immune-related adverse event (irAE). Despite receiving only one course, serum NSE levels declined significantly, and the response was judged as partial remission. Subsequently, she received one course of irinotecan and carboplatin therapy, but the therapeutic effect was poor. At the patient’s request, the regimen was changed to paclitaxel and carboplatin therapy again, which was administered for a total of eight courses. Changes in treatment corresponded with radiologic disease progression and fluctuations in serum NSE levels, as shown in **Figure 3**. However, her condition progressed, with NSE elevating to 75.9 ng/mL and CT scans revealing increased metastases in the lymph nodes and liver 10 months after the initiation of treatment for recurrence (**Figures 2C, D**). No radiologic evidence of adrenal or bone metastases was identified. Positron emission tomography–CT was planned but could not be performed because of clinical deterioration. 

At this time, psychiatric symptoms emerged. On the day metastasis progression was confirmed (defined as “day 1”), the patient began refusing treatment. The following day, the patient exhibited strange behavior, such as locking herself in the bathroom and seemingly casting a spell. On day 3, she made seemingly delusional statements, for example, “I fought the cancer and won” and “There is an evil spirit in this hospital.” When she was brought to the hospital with assistance from her husband, police, and emergency services, she was in a hallucinating state of delusion and psychomotor agitation, necessitating involuntary hospitalization for medical care by the psychiatry department.

Following admission, treatment with risperidone was initiated, leading to the alleviation of psychomotor agitation. However, delusions persisted, and the patient exhibited grandiose behavior, such as excessively expressing gratitude to hospital staff and claiming that she could win a Nobel Prize based on her ideas. Contrast-enhanced brain MRI revealed no tumor lesions or areas of abnormal signal intensity, and no findings suggestive of a pituitary mass were identified. Moreover, cerebrospinal fluid examination was within normal limits, and electroencephalography revealed generalized slow waves. In addition, pituitary-related hormones were evaluated, including thyroid-stimulating hormone, free triiodothyronine, free thyroxine, prolactin, growth hormone, and insulin-like growth factor-1, and no evidence of pituitary hyperfunction was observed. Blood tests revealed hypokalemia (2.1 mmol/L), prompting further examination, which revealed elevated ACTH (687 pg/mL) and cortisol (98.3 μg/dL) levels. Urinary free cortisol was markedly elevated at 2,870 µg/L, approximately 20–30 times the upper limit of normal. Dynamic endocrine testing, including the dexamethasone suppression test, was not performed owing to the patient’s acute psychiatric instability and rapidly progressive metastatic disease. Spot urine testing showed a urinary potassium level of 26 mmol/L, suggesting renal potassium loss in the setting of severe hypokalemia. Plasma aldosterone was <4.0 pg/mL, which did not support primary aldosteronism. These findings indicated that the hypokalemia was most likely secondary to cortisol-mediated mineralocorticoid effects associated with severe ACTH-dependent hypercortisolism. Collectively, these biochemical and clinical findings supported a diagnosis of Cushing’s syndrome associated with clinically diagnosed ectopic ACTH production, which was considered to contribute to the patient’s psychiatric symptoms. Additional dynamic endocrine tests such as inferior petrosal sinus sampling or corticotropin-releasing hormone (CRH) stimulation were not feasible due to the patient’s acute psychiatric deterioration and rapidly progressive metastatic disease. Given the challenges in treating carcinoma, metyrapone was orally administered to suppress cortisol production. On the next day after initiating this treatment, the serum cortisol level of the patient decreased to 6.6 μg/dL. Subsequently, worsening hypokalemia required transfer to the intensive care unit (ICU) for close monitoring and electrolyte correction. To prevent hypoadrenalism, hydrocortisone was also initiated. Intravenous potassium adjustment proved effective, with potassium levels improving daily.

**Figure 4:**
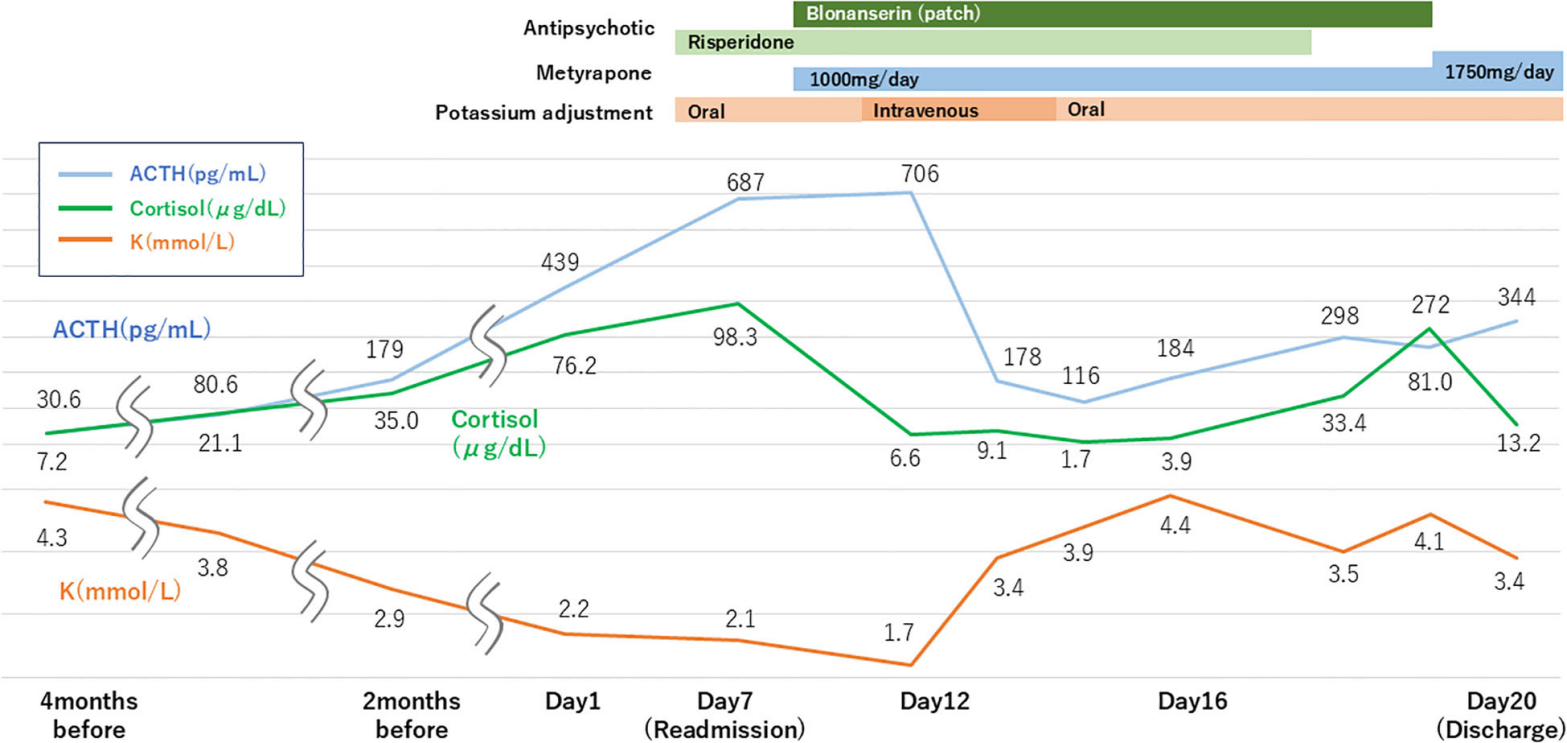
Changes in ACTH, cortisol, and potassium levels’ transition and medication details during readmission.

The patient was discharged from the ICU to the psychiatric ward on day 15. Her psychiatric symptoms improved as cortisol levels decreased; therefore, antipsychotics were discontinued. No delusional statements were observed, and the patient demonstrated insight by stating that “My previous condition was abnormal.” However, disorientation with diurnal fluctuation was noted, suggestive of terminal delirium. Considering her progressive medical condition, she was discharged from the hospital on day 20 and died at home on day 58. Details of changes in ACTH, cortisol, and potassium levels, as well as medication details, are depicted in **Figure 4**, and the progression of psychiatric symptoms during readmission is presented in **Table 1**.

**Table 1:** Progression of psychiatric symptoms.

Day 1	We showed the results of the blood test and CT scan, but she said, “I don’t want to see anything.”
Day 2	Her husband told us that her statements and behavior had been strange recently.
Day 3	She locked herself in the bathroom and cast a spell.
Day 5	Her husband called us and asked for advice on what to do.
Day 7	She was brought to the hospital by her husband with the help of the police.
Day 9	She had paranoia and told her husband that she was poisoned. She hid her symptoms and did not talk about that to the medical staff.
Day 10	Her monologue and delusion worsened, like “Thanks god, thanks god…” “I’m also a god.” She made incoherent statements, and we were unable to communicate with her.
Day 12	She could not follow instructions. She removed her catheter by herself and started to be physically restrained. → Admission to the ICU
Day 13	She had a loud monologue and disorientation.
Day 14	She could stay calm during the night. We started to be able to communicate with her again.
Day 15	She still had monologues like “Thanks god…”, but not agitation and delusion. → Discharge from ICU
Day 16	She could follow instructions and stay calm. She repeated with slight delirium.
Day 18	She had civility and orientation.
Day 20	Discharge

### 2.1 Ethics statement

This case report was conducted in accordance with the institutional ethical standards. Written informed consent was obtained from the patient for the publication of this case report and any accompanying images.

## 3 Discussion

SCCC is a rare histological subtype characterized by high malignancy and poor prognosis. The 5-year survival rate remains as low as 36.8%, even in early-stage disease (stages I–IIA) (2). Standard treatment typically includes radical hysterectomy with CCRT; however, no treatment regimen has shown a significant improvement in prognosis (3). Histologically, SCCC is classified as a small-cell neuroendocrine carcinoma, a subtype of neuroendocrine carcinoma. Tumor-derived bioactive substances can cause paraneoplastic syndromes such as the SIADH and Cushing’s syndrome (4).

Ectopic ACTH-producing tumors causing Cushing’s syndrome most commonly originate from the thoracic cavity. Notably, bronchial neuroendocrine neoplasms (NENs) and small-cell lung carcinomas are common sources. Other common causes include pancreatic NENs, thymic NENs, medullary thyroid cancer, and pheochromocytomas (5). Neuroendocrine tumors of the female reproductive tract are rare, accounting for approximately 2% of all gynecological cancers, and the uterine cervix is the most common site of occurrence (6). However, case reports of cervical neuroendocrine tumors, particularly those producing ectopic ACTH, are exceedingly rare. In a report of 11 cases of ectopic ACTH-producing tumors originating from cervical cancer, 8 cases were identified as small-cell neuroendocrine carcinomas. Notably, all patients in this series succumbed to the disease within 10 months of the diagnosis of Cushing’s syndrome (4). In a study by Di Filippo et al., nearly all cases of ectopic ACTH-producing tumors originating from SCCC presented with lung or liver metastases at the time of diagnosis of Cushing’s syndrome, suggesting that ectopic ACTH production tends to occur in more advanced stages of SCCC (7). Although ectopic ACTH production in SCCC has been reported in previous case series, the present case is distinguished by its clinical sequence, in which acute psychotic symptoms were the predominant initial manifestation and led to delayed endocrine diagnosis. **Table 2** summarizes representative previously reported cases of ectopic ACTH-producing tumors, including cervical small-cell carcinoma and selected non-gynecologic neuroendocrine tumors, to illustrate differences in initial clinical presentation. Across these reports, most cases initially presented with overt endocrine abnormalities, such as hypokalemia or Cushingoid features, whereas psychiatric symptoms were rarely the predominant initial manifestation. In contrast, the present case was characterized by acute-onset psychotic symptoms leading to psychiatric hospitalization before recognition of endocrine dysfunction, underscoring a distinct and clinically important diagnostic sequence. In the present case, the patient had multiple liver metastases and presented with clinical symptoms including psychiatric disturbances and hypokalemia. These findings align with those of a recent multicenter study by Ciftci et al., which reported that 33% of 54 patients with ectopic ACTH-producing tumors exhibited psychiatric symptoms and 67% had hypokalemia (8). Therefore, ectopic ACTH-producing tumors should be considered in the differential diagnosis of patients presenting with acute psychiatric symptoms accompanied by electrolyte abnormalities, as early recognition can substantially impact clinical management.

**Table 2:** Comparison of the present case with previously reported cases of cervical cancer with ectopic ACTH production.

Study	Initial presenting symptoms	Psychiatric hospitalization	Time to diagnosis of ectopic ACTH
Di Filippo et al., 2020 (7)	Severe hypokalemia, Cushingoid features	No	At presentation
Okumura et al., 2019* (11)	Psychosis	Yes	Several weeks
Gunther et al., 2025* (12)	First-episode psychosis	Yes	Delayed
Previously reported SCCC cases (*n* = 11, literature review) (4)	Endocrine symptoms (hypokalemia, Cushingoid features)	Rare	At or shortly after presentation
Present case	Acute-onset psychotic symptoms	Yes	Delayed after psychiatric admission

*Non-gynecologic ectopic ACTH-producing neuroendocrine tumors included for comparison of psychiatric presentation.

Cushing’s syndrome is a clinical condition characterized by various symptoms resulting from prolonged hypercortisolism. Psychiatric symptoms are recognized as key components of the clinical presentation. Depression and anxiety are the most common psychiatric manifestations of Cushing’s syndrome, whereas mania and psychosis are less frequently observed (9). The acute effects of glucocorticoids on the nervous system include neurotoxic effects mediated by excessive glutamate release, as well as suppression of neurogenesis in the dentate gyrus of the hippocampus. These mechanisms result in dysregulation of the dopamine–glutamate system, particularly within the hippocampus, which may contribute to the emergence of psychotic symptoms (1). Psychosis associated with hypercortisolism is considered to share clinical features with delirium; therefore, the presence of confusion and/or altered states of consciousness is an important factor in distinguishing it from primary manic or psychotic disorders (10). In the present case, prominent confusion and disorientation were observed, findings that further supported the diagnosis of secondary psychosis.

However, in cases of Cushing’s syndrome with acute hypercortisolism, psychosis or restlessness is more likely to occur than depression or anxiety (1). This is also observed in steroid psychosis, which is a psychiatric symptom that can occur during treatment with corticosteroids. During short-term high-dose administration of corticosteroids, euphoria or hypomanic states are more likely to occur, whereas long-term administration is more likely to result in depressive symptoms (11). In the present case, tumor metastasis and enlargement were observed immediately before the onset of psychiatric symptoms, accompanied by a rapid increase in serum cortisol levels. This finding also supports the notion that acute hypercortisolism is associated with an increased risk of developing psychiatric symptoms, such as delusions of grandeur and enhanced mood.

Treatment for ectopic ACTH production syndrome primarily involves surgery and chemotherapy targeting the underlying tumor. In the present case, despite efforts to control tumor progression with chemotherapy, the tumor progressed, prompting the initiation of metyrapone oral therapy to inhibit cortisol production. Serum cortisol levels normalized the day after metyrapone administration, and the psychiatric symptoms of the patient rapidly resolved. This is consistent with other reports, where acute psychiatric symptoms due to ectopic ACTH-producing tumors improved with normalization of serum cortisol levels (12, 13). Notably, our patient exhibited a limited response to antipsychotic medication; however, after initiating metyrapone therapy, delusions and mood elevation rapidly resolved. Overall, these findings suggest that psychiatric symptoms associated with acute hypercortisolism can achieve favorable outcomes with endocrine therapy, emphasizing the importance of differential diagnosis and appropriate treatment when addressing psychiatric symptoms in patients with cancer.

This case report has several limitations. Immunohistochemical confirmation of ACTH production and neuroendocrine markers in the recurrent lesion could not be performed due to limited tissue availability. Dynamic endocrine testing, including inferior petrosal sinus sampling or CRH stimulation, was not feasible because of the patient’s acute psychiatric deterioration and rapid progressive disease. Furthermore, pathological or molecular re-verification of the recurrent tumor was not possible. Therefore, the diagnosis was based on converging clinical, biochemical, radiological, and therapeutic findings, and is described as clinically diagnosed ectopic ACTH production.

In this case, the patient underwent radical trachelectomy followed by chemotherapy alone, following her strong desire to preserve fertility. However, considering her high-risk features, specifically FIGO (International Federation of Obstetrics and Gynecology) stage IB1 disease and lymphovascular invasion, we believe that more aggressive initial treatments, such as CCRT, would have been more appropriate. Initially, she was hospitalized with suspected psychiatric illness but was later diagnosed with organic psychosis secondary to an endocrine disorder. This case highlights the importance of recognizing that psychiatric symptoms in patients with cancer may arise from underlying endocrine abnormalities. Patients with SCCC are at risk of paraneoplastic endocrine syndromes such as ectopic ACTH production, which can manifest as neuropsychiatric symptoms.

## 4 Conclusion

This case highlights that SCCC can present psychiatric symptoms due to ectopic ACTH production. SCCC may be associated with clinically diagnosed ectopic ACTH production, which can manifest as acute psychiatric symptoms in advanced disease. Such symptoms may indicate advanced disease and should prompt evaluation for endocrine abnormalities. Early recognition and treatment of paraneoplastic Cushing’s syndrome can improve outcomes, and clinicians should consider this possibility, especially in neuroendocrine tumors like SCCC.

## Data Availability

The raw data supporting the conclusions of this article will be made available by the authors, without undue reservation.

## References

[1] Pivonello R Simeoli C De Martino MC Cozzolino A De Leo M Colao A . Neuropsychiatric disorders in Cushing’s syndrome. Front Neurosci. (2015) 9:129. doi: 10.3389/fnins.2015.00129, PMID: 25941467 PMC4403344

[2] Cohen JG Kapp DS Shin JY Urban R Sherman AE Chen LM . Small cell carcinoma of the cervix: treatment and survival outcomes of 188 patients. Am J Obstet Gynecol. (2010) 203:347. doi: 10.1016/j.ajog.2010.04.019, PMID: 20579961

[3] Satoh T Takei Y Treilleux I Devouassoux-Shisheboran M Ledermann J Viswanathan AN . Gynecologic Cancer InterGroup (GCIG) consensus review for small cell carcinoma of the cervix. Int J Gynecol Cancer. (2014) 24:S102–8. doi: 10.1097/IGC.0000000000000262, PMID: 25341572

[4] Patel H Kishlyansky D Köbel M Alshamma Z Glaze S Ghaznavi S . Cervical small cell neuroendocrine carcinoma with ectopic ACTH secretion and acute pancreatitis. J Endocr Soc. (2023) 7:1-8. doi: 10.1210/jendso/bvac192, PMID: 41268020

[5] Ragnarsson O Juhlin CC Torpy DJ Falhammar H . A clinical perspective on ectopic Cushing’s syndrome. Trends Endocrinol Metab. (2024) 35:347–60. doi: 10.1016/j.tem.2023.12.003, PMID: 38143211

[6] Salvo G Gonzalez Martin A Gonzales NR Frumovitz M . Updates and management algorithm for neuroendocrine tumors of the uterine cervix. Int J Gynecol Cancer. (2019) 29:986–95. doi: 10.1136/ijgc-2019-000504, PMID: 31263021

[7] Di Filippo L Vitali G Taccagni G Pedica F Guaschino G Bosi E . Cervix neuroendocrine carcinoma presenting with severe hypokalemia and Cushing’s syndrome. Endocrine. (2020) 67:318–20. doi: 10.1007/s12020-020-02202-x, PMID: 31970585

[8] Ciftci S Yilmaz N Selcukbiricik OS Hekimsoy Z Canpolat AG Topsakal S . Comparison of clinical, hormonal, pathological and treatment outcomes of ectopic Cushing’s syndrome by sex: results of a multicenter study. Endocrine. (2024) 86:1148–55. doi: 10.1007/s12020-024-04004-x, PMID: 39287756

[9] Jeffcoate WJ Silverstone JT Edwards CR Besser GM . Psychiatric manifestations of Cushing’s syndrome: response to lowering of plasma cortisol. Q J Med. (1979) 48:465–72. 542586

[10] Keshavan MS Kaneko Y . Secondary psychoses: an update. World Psychiatry. (2013) 12:4–15. doi: 10.1002/wps.20001, PMID: 23471787 PMC3619167

[11] Warrington TP Bostwick JM . Psychiatric adverse effects of corticosteroids. Mayo Clin Proc. (2006) 81:1361–7. doi: 10.4065/81.10.1361, PMID: 17036562

[12] Okumura T Takayama S Nishio SI Miyakoshi T Noguchi T Kobayashi T . ACTH-producing thymic neuroendocrine tumor initially presenting as psychosis: A case report and literature review. Thorac Cancer. (2019) 10:1648–53. doi: 10.1111/1759-7714.13099, PMID: 31187563 PMC6610259

[13] Gunther M Jiang S . First-episode psychosis and Cushing syndrome. Prim Care Companion CNS Disord. (2025), 24cr03886. doi: 10.4088/PCC.24cr03886, PMID: 40163351

